# Patient-reported experience measures (PREMs) in outpatient psychiatry – is there an association to perceived discrimination and devaluation?

**DOI:** 10.3389/fpsyt.2024.1378487

**Published:** 2024-04-17

**Authors:** Janina Billian, Lukas Imfeld, Carl B. Roth, Julian Moeller, Undine E. Lang, Christian G. Huber

**Affiliations:** ^1^ Universitäre Psychiatrische Kliniken (UPK) Basel, Klinik für Erwachsene, University of Basel, Basel, Switzerland; ^2^ Faculty of Psychology, Division of Clinical Psychology and Epidemiology, University of Basel, Basel, Switzerland; ^3^ Institute for Evaluation Research, Basel, Universitäre Psychiatrische Kliniken (UPK) Basel, University of Basel, Basel, Switzerland

**Keywords:** social stigma, patient satisfaction, self report, mental health, community mental health services, ambulatory care, health services research

## Abstract

Patient-Reported Experience Measures (PREMs) are gaining significance in the field of psychiatry, with patient satisfaction being a key measure. However, it is unclear if patient satisfaction in psychiatry is influenced by variables outside the treatment setting. This brief report thus examines the possible impact of perceived discrimination and devaluation (PDD) on patient satisfaction in the psychiatric outpatient setting. Data from 1,126 individuals who were undergoing or had recently completed treatment at 15 outpatient centers of the Psychiatric University Clinic in Basel, Switzerland, was analyzed. Patient satisfaction was assessed using the Münsterlingen Patient Satisfaction Questionnaire (MüPF), and perceived stigma was measured with the Perceived Discrimination Devaluation Scale. The results revealed a positive small effect size relationship between MüPF and PDD, suggesting that patients who perceived less stigma report higher treatment satisfaction. This relationship may affect most ratings, with the total MüPF score remaining relatively robust against this potential influence. Linear regression analysis indicated that a one-unit increase in PDD score could lead to a maximum change of 1.8 points on the 7-point Likert scale for the MüPF item correlating highest with PDD and 0.4 points on the total MüPF score. These findings highlight the importance of considering perceived stigma when evaluating patient satisfaction with psychiatric outpatient treatment. Future research should investigate associations between stigma, patient satisfaction, treatment outcome, as well as other external factors that may influence patient satisfaction in psychiatric settings.

## Introduction

The quality of healthcare services is not solely determined by medical expertise and technological advancements; it also heavily relies on the patients’ experience and satisfaction. Modern psychiatry aims to provide comprehensive, person-centered care that takes into account the patient’s individual wishes, goals and needs (1–5). There is evidence to suggest that patient experience is positively associated with self-rated and objectively measured health outcomes, patient safety, clinical effectiveness, and adherence to recommended clinical practice which supports the argument for including patient experience as a key component of healthcare quality (6).

There is an increasing focus on collaborative treatment, in which the patient is actively involved in decisions about their treatment (6–9). Hence, patient-reported experience measures (PREMs) are becoming increasingly important in medical care in general and in psychiatry in particular as they are designed to measure patient experience with care, i.e., whether and how the patient experienced certain processes of care and the quality of that experience (10–13).

One of the most important PREMs for evaluating mental healthcare systems is patient satisfaction with multiple aspects of treatment, including, e.g., the patient’s subjective experience of relationship with the treatment team, receiving adequate information about the treatment and being able to influence treatment decisions (14, 15). However, it is currently unclear if self-reported patient satisfaction may be systematically influenced by factors outside the treatment setting (15–17).

Previous research suggests that patient satisfaction ratings are influenced by stigmatization (18). Mental health stigma leads to impaired self-esteem and creates significant barriers to seeking help and accessing appropriate treatment, exacerbating the already challenging experiences faced by those with mental health conditions (19–26). It has been shown to increase co-morbid depression and to influence the course of treatment and dropout rates in psychiatric settings (27–34). Perceived stigma of individuals with mental illness encompasses the negative beliefs and stereotypes assigned to them by society, often resulting in discrimination and social exclusion (35). In particular, the experience of discrimination and devaluation, whether explicitly or implicitly expressed, often lead to negative healthcare experiences, decreased patient involvement, and ultimately affect overall satisfaction.

Thus, the aim of the current analysis is to investigate whether perceived discrimination and devaluation (PDD) might be connected with patient satisfaction in the psychiatric outpatient setting. Based on the current literature, we hypothesized that higher perceived discrimination might be related to lower satisfaction with treatment.

## Method

### Participants

The survey was directed at individuals who were currently undergoing treatment or had recently completed treatment at one of 15 outpatient treatment centers of the Psychiatric University Clinic in Basel, Switzerland, in 2018 and 2021.

These 15 outpatient services offer a range of treatment focuses, with some specializing in specific diagnoses such as substance abuse disorders, psychotic disorders, or personality disorders, while others provide treatment for various types of mental illnesses.

The UPK Basel routinely conducts anonymous patient satisfaction surveys as part of its quality management procedures. No ethics committee vote was necessary for the analysis and publication of this anonymously collected routine quality management data. This was confirmed by the responsible ethics committee (Ethics Committee of Northwestern Switzerland; EKNZ; Req-2023-01405).

### Procedure

Participants were sent self-report questionnaires via standard mail and were invited to participate voluntarily. They were informed about the purpose and methodology of the survey and assured of the confidentiality and anonymity of their responses. A stamped envelope, addressed to the Institute for Evaluation Research, Basel, was enclosed. In 2021, participants could fill out the questionnaires additionally digitally. The questionnaires in digital format were available either by scanning a QR code or via an Internet link to the Unipark survey site.

### Assessments

Demographic information asked for included age, gender, and nationality of the participants. Furthermore, characteristics of course of treatment, e.g. when the person had started their treatment, whether they had contacted the outpatient service themselves, and whether they had finished treatment at the time of the survey, were requested.

Patient satisfaction in the outpatient setting was assessed using the *Münsterlingen Patient Satisfaction Questionnaire* (in German: “Münsterlinger Fragebogen zur Patientenzufriedenheit”; MüPF). This PREM questionnaire has been developed by the MüPF Benchmark Group in 2008/2009 in cooperation with the Institute for Evaluation Research, Basel. It contains 26 items and is intended to reflect the following treatment aspects: Admission, information, involvement, organization, discharge, security, medication, dignity, contact with other patients, partnership with health caregivers, overall satisfaction and recommendation of treatment site (for an overview: 12, 36, p. 26). For two items, there was no data from 2018 because the item wording was adjusted for the survey from 2021. This affects the items “Change of therapist was well prepared and carried out.” and “I had no inhibitions about asking my doctor/psychologist questions.” Each aspect is rated on a 7-point Likert scale ranging from 1 (“does not apply at all”) to 7 (“fully applies”). There is a residual response category ‘not answerable’ for all items. Two additional open questions offer a free field for participants to describe particularly positive aspects of treatment, disturbances and suggestions for improvement in the outpatient treatment center. An overall MüPF mean score was calculated by adding the 26 item values and dividing them by their number.

The *Perceived Discrimination Devaluation Scale* (PDD) consists of twelve items measuring the perception of devaluation and discrimination toward persons with mental illness in the general population (37). Responses indicate the level of agreement with each statement, rated on a 5-point Likert scale ranging from 1 (“does not apply”) to 5 (“applies fully”). A high level of perceived stigma against people with mental illness is indicated by disagreement with six positively poled items (e.g. “Most employers will hire a former mental patient if he or she is qualified for the job.”) and agreement with six negatively poled ones (e.g. “Most employers will pass over the application of a former mental patient in favor of another applicant.”). The scale demonstrated sufficient global internal consistency of *α* = 0.84 (38). For the purpose of this study, the German translation of the PDD was used (39). For the twelve items of the PDD, the six negatively worded items were reversed so that higher scores corresponded to lower perceived stigma. An overall PDD mean score was calculated by summing the values of the twelve items and dividing by twelve. A low PDD score indicates that the individual perceives the extent of stigmatization of individuals who are or have been in psychiatric treatment as strongly pronounced.

### Data analysis

The data collected was imported into the statistical program IBM SPSS Statistics 27.0 (40). To examine the correlation between patient satisfaction items and perceived discrimination and devaluation of people with mental illness, Pearson’s correlation analysis was performed. This statistical technique assesses the strength and direction of the linear relationship between two continuous variables. A significance level of *p* < 0.05 was used to determine the statistical significance of the correlation coefficient.

A Bonferroni correction for multiple correlations was applied in order to reduce the likelihood of Type I errors and account for the increased risk of falsely detecting significant associations when conducting multiple statistical tests (41).

The number of responses varied for each variable, as patients could also choose the response that they were unable to answer a specific question. Any missing values were excluded pairwise from analysis.

To estimate the extent to which patient satisfaction ratings could be influenced by perceived stigmatization, we conducted linear regression analyses using the PDD score as the predictor variable for both the total MüPF score and the MüPF item with the highest correlation with the PDD score, as a means to predict patient satisfaction.

## Results

The final sample consisted of 1,126 individuals, of whom 525 took part in 2018 and 601 in 2021. 535 (47.5%) were female. The age of participants ranged between 18 and 99 years with a mean (*m*) of 46.8 and a standard deviation (*SD*) of 16.8.

With a theoretical range from 1 (worst) to 7 (best), the MüPF total average in the population was *m* = 4.6 with a standard deviation of *SD* = 1.2. The PDD total average with a theoretical range from 1 (low) to 5 (high) was *m* = 3.2 (*SD* = 0. 8).

The MüPF and PDD mean scores were correlated significantly (*r* = 0.067, *p* = 0.03) with a small effect size. This suggests that – in general – patients with less perceived stigma also tended to have a higher treatment satisfaction and vice versa.

**Table 1** depicts the correlations between all of the patient satisfaction items and the PDD score. The single item aimed to measure general treatment satisfaction (“Overall, I am satisfied with my treatment”) showed a significant positive correlation (*r* = 0.118; *p* < 0.001) with a small effect size. In addition, the majority of correlations between the 26 individual items of the MüPF scale and their PDD score were statistically significant. Overall, correlations with small effect sizes were found, with a maximum of *r* = 0.211.

**Table 1:** Pearson correlations between patient satisfaction with outpatient treatment (MüPF) and perceived discrimination and devaluation (PDD).

Patient satisfaction with treatment	*r-value*
The service is easy to reach by phone.	.106^*^
I quickly got my initial appointment.	.124^*^
I was received in a friendly manner.	.118^*^
I was able to explain my situation sufficiently in the initial consultation.	.104^*^
Changes of therapists were well prepared and carried out.	.098
I felt respectfully treated by the service staff.	.074^*^
When I am in need, I know where to turn.	.072^*^
I trust the people who treat me.	.096^*^
I had no inhibitions about asking my doctor/psychologist questions.	.126^*^
My illness was explained to me in a comprehensible way.	105^*^
Treatment goals were agreed with me.	.114*
I was able to influence the planning of my treatment.	.142^*^
The effects of the medication and possible side effects were explained to me in an understandable way.	.092^*^
I was able to influence the medical treatment.	.105^*^
The treatment staff at the service had enough time to talk to me.	.092*
I was supported and accompanied in my search for other help (e.g. offices, self-help groups).	.078*
I had the feeling that I had competent specialists as discussion partners.	.086*
The cooperation between my relatives and those treating me met my needs.	.081
How helpful did you find the collaboration with your doctor?	.138^*^
How helpful did you find the collaboration with your psychologist?	.166^*^
How helpful did you find the cooperation with your nurse?	.136^*^
How helpful did you find the cooperation with your social worker?	.154^*^
How helpful did you find the collaboration with your other therapists?	.211^*^
The treatment helps me to better deal with my problems.	.135^*^
Overall, I am satisfied with my treatment.	.118^*^
I would recommend this treatment.	.079^*^

*The correlation is significant at p < 0.05 (2-sided), Bonferroni corrected. The correlation highlighted in bold remained significant after the Bonferroni correction.

Two correlations did not reach significance. Whether changes of therapists were well prepared and carried out and whether cooperation between relatives and therapists met patients’ needs did not seem to be connected with PDD.

To assess the extent to which patient satisfaction ratings could be influenced by perceived stigmatization, we conducted linear regression analyses using the PDD score as the predictor variable for both the total MüPF score and the MüPF item with the highest correlation with the PDD score, as a means to predict patient satisfaction.

As a result of the Bonferroni correction, 12 correlations remained significant, while 14 correlations were found to be nonsignificant. The correlation highlighted in bold remained significant after the Bonferroni correction, as shown in **Table 1**.

A linear regression analysis with the PDD score as the predictor and the MüPF item with the highest correlation with PDD (i.e. “How helpful did you find the collaboration with your other therapists, e.g. physiotherapists, therapists for music, occupational therapy etc.?”) was calculated. The regression model accounted for approximately 4.1% of the variance in that MüPF item (*R²* = 0.041) with an increase in satisfaction of 0.458 units (*β* = 0.458, *p* < 0.001) for every unit increase in the PDD score. Thus, with a maximum scale difference of four points on the PDD, the maximal change on the MüPF item would be 1.8 points on the 7-point Likert scale.

Using the same approach with the MüPF total score as the dependent variable generated a regression model that accounted for approximately 0.4% of the variance (*R²* = 0.004). For each one unit increase in the PDD score ranging from 1 to 5, overall treatment satisfaction increased by an average of 0.1 units (*β* = 0.100, *p* = 0.028) on the MüPF scale ranging from 1 to 7. Given that the maximum scale difference on the PDD is four points, the maximum change on the MüPF total score would be 0.4 points on the 7-point Likert scale.

## Discussion

This study is – to the authors’ knowledge – the first to examine the connection between perceived stigma, a prevailing problem for persons with mental health issues, and the highly relevant PREM patient satisfaction in the psychiatric outpatient setting. With 1,126 participants, a large patient sample was available for analysis.

In accordance with our hypotheses, we found that total satisfaction, the single item measuring general satisfaction, and most individual items showed significant associations with small effect size between 0.10 and 0.29 (42), indicating higher patient satisfaction in patients with lower perceived discrimination.

Although individual patients satisfaction items could be influenced to a relevant degree with a maximal change of 1.8 on a 7-point Likert scale, the patient satisfaction questionnaire in general seem relatively robust regarding this influence with a maximal change of 0.4.

Due to the nature of the data and analyses, no statements regarding causality or direction of effects are possible. In theory, patients with high perceived stigmatization might be biased to experience treatment settings and interactions in a more negative way. This might lead to a more negative treatment satisfaction that cannot be ascribed to the treatment setting itself. However, other interpretations are possible. For example, lower stigmatization could be connected with the ability to benefit more from the treatment, leading to higher treatment satisfaction. While in the first scenario, treatment satisfaction would be rated differently because of non-treatment related factors, this would not be the case in the second scenario.

Patient satisfaction is a complex concept that is influenced by a variety of factors both within and outside of the therapeutic setting. While patient experience and health status play a role in shaping satisfaction with the health-care system, Bleich et al. (16) argue that broader societal factors may largely account for the unexplained portion of satisfaction. Furthermore, Stamboglis and Jacobs (17) highlight the significant impact of patient characteristics, treatment type, care continuity, and service integration on patient satisfaction. Additionally, Batbaatar and colleagues (15) emphasize the importance of health providers’ interpersonal care quality in determining patient satisfaction. These findings suggest that variables such as social support within and outside of psychiatric settings, patient expectations, patient’s age and gender, financial resources, employment status, and physical health needs may also play a crucial role in shaping patient satisfaction. It is clear that a comprehensive understanding of patient satisfaction requires consideration of a wide range of factors both within and outside of the therapeutic setting.

Certain limitations of the current study have to be taken into consideration. Data were collected at only one study center. Future research has to show if the reported correlations can also be found in other samples and if the findings can be replicated. Furthermore, other variables outside the therapeutic setting like the capacity for introspection or the tendency to internalize or externalize negative and positive events could probably be connected to patient satisfaction and should be explored in further research.

In summary, the results demonstrate a significant relationship of small effect size between perceived discrimination and devaluation and patient satisfaction in individuals with mental illness. This relationship may impact individual ratings, but the total score remains relatively stable against this influence. These findings have important clinical implications for healthcare practice in the psychiatric outpatient setting, emphasizing the need to address stigma and discrimination in mental health care to enhance patient satisfaction and outcomes. Patients with lower perceived discrimination tend to display higher satisfaction with their treatment, particularly in areas such as collaboration with healthcare providers, ability to influence treatment planning, and overall treatment satisfaction. Healthcare providers should be mindful of the impact of stigma on patient experience, ensuring patients feel respected, valued, and involved in their treatment decisions.

Thus, future research is needed to further elucidate the nature of the association of stigma and patient satisfaction, and to examine if they may lead to biased satisfaction ratings. Moreover, other external variables that may affect patient satisfaction beyond the therapeutic setting, should be investigated, such as the capacity for introspection and the tendency to internalize or externalize events. This comprehensive understanding of patient satisfaction is essential for delivering high-quality, patient-centered care in psychiatric settings.

## Data availability statement

The raw data supporting the conclusions of this article will be made available by the authors, without undue reservation.

## Ethics statement

The studies involving humans were approved by Ethics Committee of Northwestern Switzerland. The studies were conducted in accordance with the local legislation and institutional requirements. Written informed consent for participation was not required from the participants or the participants’ legal guardians/next of kin in accordance with the national legislation and institutional requirements.

## Author contributions

JB: Writing – original draft, Formal analysis. LI: Writing – review & editing. CR: Writing – review & editing, Conceptualization. JM: Formal Analysis, Writing – review & editing. UL: Writing – review & editing. CH: Writing – original draft, Formal analysis, Conceptualization.
